# PEGylation of Truncated Streptokinase Leads to Formulation of a Useful Drug with Ameliorated Attributes

**DOI:** 10.1371/journal.pone.0155831

**Published:** 2016-05-18

**Authors:** Pooja Sawhney, Keya Katare, Girish Sahni

**Affiliations:** Department of Molecular Biology and Protein Science and Engineering, CSIR-Institute of Microbial Technology, Sector 39-A, Chandigarh, India; Tecnologico de Monterrey, MEXICO

## Abstract

Streptokinase (SK) remains a favored thrombolytic agent in the developing world as compared to the nearly 10-fold more expensive human tissue-plasminogen activator (tPA) for the dissolution of pathological fibrin clots in myocardial infarction. However, unlike the latter, SK induces systemic activation of plasmin which results in a greater risk of hemorrhage. Being of bacterial origin, it elicits generation of unwanted antibody and has a relatively short half-life *in vivo* that needs to be addressed to make it more efficacious clinically. In order to address these lacunae, in the present study we have incorporated cysteine residues specifically at the N- and C-termini of partially truncated SK and these were then PEGylated successfully. Some of the obtained derivatives displayed enhanced plasmin resistance, longer half-life (upto several hours), improved fibrin clot-specificity and reduced immune-reactivity as compared to the native SK (nSK). This paves the way for devising next-generation SK-based thrombolytic agent/s that besides being fibrin clot-specific are endowed with an improved efficacy by virtue of an extended *in vivo* half-life.

## Introduction

Streptokinase (SK) is an affordable drug in resource-limited countries for treatment of circulatory disorders like ischemic stroke, myocardial infarction and pulmonary embolism. It is secreted by beta-hemolytic bacteria e.g. *Streptococcus equisimilus* [1]. Being of non-human origin, it can trigger an immune response which may cause allergic and hemorrhagic reactions [2]. Besides high antigenicity, it has other shortcomings like short half-life and rapid kidney clearance. Nonetheless, as a plasminogen activator, it exhibits efficiency equivalent to that of relatively expensive tissue-plasminogen activator (tPA) or its improved derivatives [3]. Streptokinase activates plasminogen (PG) through a complex pathway. Unlike other PG activators which directly act on their substrate (plasminogen), SK interacts with PG (zymogen) which consecutively undergoes a complex, poorly understood conformational rearrangement, and forms an active, highly substrate-specific SK.Plasmin(ogen) “activator” complex. This complex then cleaves a scissile peptide bond between Arg^561^-Val^562^ of PG and this results in generation of plasmin (PN), a non-specific proteolytic enzyme, which catalyses dissolution of fibrin clots [4].

The structural-functional inter-relationship of SK with PG, in binary and ternary complexes, has been elegantly elucidated in recent years [5–12]. SK follows two distinct pathways for PG activation [4, 13–15]. In pathway 1, SK binds with PG and the entailing molecular rearrangements result in formation of a non-proteolytically active zymogen complex which displays a near-identical amidolytic activity as exhibited by free plasmin. This complex undergoes conformational changes and gets converted to a fully-functional SK-human plasmin (SK.HPN) active complex. It has been postulated that the activation of zymogen involves conformational changes either by proteolytic release of Val 562 of plasminogen or due to binding of SK. In fact, activation of zymogen has been demonstrated by amidolytic assays. Several investigations have supported the Bode and Huber 'molecular sexuality theory' considering the importance of the N-terminus (Ile1) amino acid of SK during plasminogen activation. Deletion of Ile1 at N-terminal of SK impairs its potential of creating an active site in plasminogen via a non-proteolytic mechanism [16]. Crystal structure data of SK also validated the role of N-terminal of SK in zymogen activation, thereby supporting the so-called 'molecular sexuality theory' [12]. X-ray crystal structure of SK.μPN complex have revealed the various covalent and non-covalent interactions involved in maintenance of the binary complex and also the selective substrate-binding exosites as deduced earlier from the earlier biochemical and biophysical binding studies [5, 7, 9, 10]. The alpha domain of SK was observed to potentially participate in substrate-recognition along with regions of the beta domain that are not implicated in activator complex formation *per se*. The gamma domain of SK binds in vicinity of activation loop of the 'partner' μPG and contributes in evoking conformational changes in the PG molecule leading to the activation of zymogen of the complex. During this process, the N-terminal region of SK has a particularly important role. In the second pathway (Pathway 2), SK directly binds with plasmin forming an active SK.HPN complex which then accelerates the conversion of substrate PG into PN. In fact, plasmin has a higher binding affinity for SK as compared to plasminogen. Numerous investigations indicate that deletion of upto 16 amino acids from the N-terminal end and of upto 27 amino acids from the C-terminal of SK do not significantly impair its biological activity [17–19].

Diverse approaches have been employed for obviating the shortcomings such as antigenicity, short half-life of therapeutically important proteins. These include selective amino-acid modifications [20] and conjugation with various compounds such as peptides [21], albumin [22, 23] and carbohydrate-moieties like poly-ethylene glycol (PEG) [24, 25]. Among these, PEGylation is reported to be quite promising [26, 27]. In fact, more than ten PEGylated therapeutics have already received approval of US FDA and many more are in the pipe-line [26, 28, 29]. Investigations on PEGylation of SK have been conducted earlier [30]. In these studies, low molecular weight PEG moieties (2–8 KDa) were introduced rather non-specifically into the polypeptide by lysyl side-chain modification using carbodiimide mediated condensation reaction. Under such conditions, the bioactivity was observed to be progressively compromised as the number of PEG groups increased. Previously, we attempted to design an improved full-length streptokinase by conjugating PEG-moieties within each domain or into two and in all the three domains of native SK [31, 32]. Mono and bi-PEGylated derivatives did exhibit higher activities, much improved half-life and reduced immune-reactivity. However higher order of PEGylation was seen to compromise the activity significantly. Even when the bioactivity was reasonably retained along with a suitably increased (and potentially useful) *in vivo* half-life, we could not obtain any derivative with an improved fibrin clot specificity, a much coveted clinical trait. Despite the improvement in half-life, PEGylation of full-length SK still leaves some uncovered immunological hot-spots and epitopic regions which can evoke immune response. Therefore, shortcomings of full-length SK prompted us to re-design PEGylated truncated-SK molecules with diminished immunogenicity. Previously, truncated SK derivatives with native-like activities [17–19] with reduced epitopic regions at their N and C-terminii have been reported. Clot-specificity is another important parameter that is desirable for an efficient thrombolytic drug. SK generates systemic activation in circulation and degrades fibrinogen in a speedy manner. Therefore, SK derivatives devoid of systemic activation of PG are required for superior therapeutic usage. In the present investigation, we have engineered truncated SK constructs with an additional cysteine residue incorporated at either the N- or C-termini for site-specific thiol chemistry-based PEG-conjugation. Employing this approach, we have successfully obtained and characterized PEG-SK preparations with desirable combination/s of fibrin clot specificity, reduced immunogenicity and clinically relevant *in vivo* survival rate having bioactivity comparable to that of native SK.

## Materials and Methods

### Materials

*SK* gene was cloned in T7 RNA polymerase promoter-based expression vector, pET-23d [7] and was transformed in *Escherichia coli* BL21 (DE3) strain obtained from Novagen Inc. (Madison, WI). Restriction endonucleases, Thermostable DNA polymerase (*pfu* Turbo^TM^), T4 DNA ligase and other DNA modifying enzymes were purchased from New England Biolabs (Beverly, MA). Oligonucleotide primers were provided by Integrated DNA technologies (IDT), Coralville, Iowa. PCR-extraction and purification of DNA were carried out with kits available from Qiagen GmbH (Germany). Glu-plasminogen was either procured from Roche Diagnostics GmbH, Germany or purified from human plasma by affinity chromatography in the presence of inhibitors etc. [33] DEAE Sepharose^™^ (Fast Flow), Phenyl-Agarose 6XL and Sephacryl high resolution gel filtration media were purchased from GE-Healthcare. Methoxy-PEG maleimide of 10KDa and 20 KDa MW were products of JenKem Technology, USA. All the reagents used were of highest analytical grade available.

### Animal Study

The study on animals was carried out strictly in accordance with the ethical guidelines of the Institute (Institute of Microbial Technology) and approved by Institutional Animal Ethics Committee (IAEC) vide Approval number IAEC/11/5.

### Design and construction of SK derivatives

Native *SK* gene, from *Streptococcus equisimilus* H46A strain, earlier cloned in pET 23d vector[7] was used for deletion and substitution mutations. The nSK contains 414 amino acids but the truncated SK has only 1–383 amino acid residues and has been denoted as SK1-383. The vector (pET 23d) also contains ampicillin resistance gene which was used for the selection. Cysteine residues were incorporated at the desired terminal/s (N or C-terminal) and represented as SK1-383N and SK1-383C respectively, with commercially available Quick-change® Mutagenesis kit (Agilent Technologies, USA) using two complementary primers which contained the desired mutation. *pfu turbo* enzyme was used to replicate both plasmid strands with high fidelity. *Dpn I* enzyme, which cleaves methylated and hemi-methylated DNA, was used to digest parental plasmid. The nicked plasmid was transformed into *E*.*coli* XL1-Blue chemical competent cells. Positive clones with the desired mutation were then confirmed by DNA sequencing. Confirmed plasmids were transformed into *E*. *coli* BL21DE3 cells for expression of the protein.

### Expression and purification of streptokinase and its mutants

Streptokinase and its cysteine mutants were over-expressed as inclusion bodies in *E*.*coli* strain BL21DE3 cells under the control of T7 phage RNA polymerase promoter after induction with isopropyl-1-thio-β-D-galactopyranoside and subsequently purified with slight modifications as described earlier [7]. The bacterial culture (1L) was pelleted by centrifugation at 8000 rpm for 15 min at 4°C and lysed after washing with cold 80 ml STE buffer containing 100 mM NaCl, 10 mM Tris-HCl (pH 7.5), 1 mM EDTA (pH 8). Cell pellet was then suspended in 33 ml cold STE buffer containing 100mM NaCl, 10 mM Tris-HCl (pH 7.5), 1 mM EDTA (pH 8) and sonicated for a total time of 50 min with 30 sec on and 30 sec off pulse rate. The cell lysate was centrifuged at 12,500 rpm for 20 min at 4⁰C to obtain inclusion body (IB) pellet. IB was further washed twice with 20 ml STE buffer containing 2 M urea and 0.5% Triton X- 100. The pellet was then centrifuged at 12500 rpm for 15 min at 4⁰C and dissolved in 20 mM phosphate buffer containing 8 M Urea and and incubated for 1 hr with gentle shaking. The inclusion body contain denatured protein in 8M urea was centrifuged to recover the protein in supernatant. The protein was diluted 15-fold in equilibration buffer containing 0.5 M NaCl, 20 mM phosphate buffer, pH 7.2 and 0.1 mM DTT. The diluted protein was loaded over a pre-equilibrated phenyl agarose hydrophobic interaction chromatography (HIC) column and eluted with water after washing the column with 3–4 bed volume equilibration buffer at 4°C. Protein fractions were quantified by Bradford estimation and checked for purity on SDS-PAGE gel. All HIC fractions that contained the pure protein were pooled and loaded over an equilibrated DEAE-sepharose column (GE-Healthcare). The column was then washed with an equilibration buffer containing 20 mM phosphate buffer (pH 7.5) to remove unbound protein followed by elution of the desired protein with an elution buffer containing 1 M NaCl in 20 mM phosphate buffer (pH 7.5) in a linear gradient manner. Protein fractions were collected and examined quantitatively by Bradford [34] and qualitatively by SDS-PAGE gel analysis.

### SDS-PAGE and MALDI-TOF analysis of PEGylated and unPEGylated SK constructs

All PEGylated and unPEGylated derivatives were checked for their purity and conversion (unPEGylated to PEGylated form) on the SDS-PAGE gel. For this, 5ug of the protein (PEGylated or unPEGylated) was mixed in 5X loading dye (reducing and non-reducing dye separately) and separated on 7.5% polyacrylamide gel. Gel was run slowly in 1X Tris-glycine buffer at 25 mA for 2 hours for fine separation. When gel was over, it was placed with coomassie staining solution (Bio-Rad) for 1h followed by repeatedly washing in destaining solution to clearly visualize the sharp bands. Reference protein ladder (GE-Healthcare) was used to determine the exact protein size. Although, the accurate determination of the molecular weights were determined by MALDI-TOF. For MALDI-TOF analysis, 1 mg/ml protein was desalted in water on 1 ml column containing G-25 Sephadex media. The desalted proteins were analysed on an AB SCIEX instrument (model-AB SCIEX TOF/TOF^TM^ 5800) for their accurate molecular weights.

### Human Plasminogen (HPG) activation assays with and without plasmin by native SK and its cysteine mutants

A one-stage colorimetric assay was used to determine the kinetics of activation of HPG by native SK (nSK) and its cysteine mutants [35]. Continuous change in absorbance was measured at 405 nm with increasing time in a BioTek^TM^ Elisa Reader (Model Gen 5 1.08) at 25°C. Activities of the activators were also measured by incubating plasmin with 1 nM and 2 nM concentrations along with HPG and Chromozyme ^®^ PL in the assay. The activities were calculated from the slopes of the progress curves by plotting graphs of change in absorbance/time-square [35, 36].

### Amidolytic activation of HPG/uPG by nSK/PEG-SK variants at 37°C and 4°C

HPG/μPG (final concentration, 5μM) was incubated with native streptokinase (nSK) /PEGylated SK (final concentration 5.5 μM) in a 50 mM Tris(hydroxymethyl aminomethane)-HCl buffer, pH 7.5 containing 0.5% bovine serum albumin. Then the complex was transferred into the well containing assay buffer having 50 mM Tris-HCl, pH 7.5, 100 mM NaCl along with 1 mM Chromozyme ^®^PL to a final concentration of 10 nM. The time-course of amidolytic activity generation was measured by recording absorbance at 405 nm with time [5, 16].

### Determination of clot-lysis by native SK and its PEGylated variants

Fibrinolytic activity of nSK and its PEGylated variants were determined as described previously [37] with slight modifications. Briefly, a 100 μl clot was prepared by incubating 12 μM fibrinogen (human, Sigma, USA), 50 nM thrombin (human, Calbiochem, La Jolla, CA), 25 mM CaCl_2_ and 100 nM Glu-PG (Roche Diagnostics GmbH, Germany) at 37°C for 1 hour. Fibrinogen was dissolved in buffer containing 40 mM Tris-HCl buffer (pH 7.4), 75 mM NaCl and 0.01% Tween 20. Subsequently, the different concentrations of SK and its variants, i.e., 5 nM to 50 nM were added to the clot and fibrinolysis was observed by change in fibrin opacity at 405 nm. The time point for 50% and 100% clot-lysis was calculated from the absorbance vs time data.

### Fibrinogen estimation in plasma containing nSK or PEGylated SK

Effect of activators on the level of fibrinogen present in circulating plasma was determined by measuring total fibrinogen concentration in plasma in the presence of activators at various time-intervals. 25 nM concentration of nSK and PEGylated SK constructs were independently incubated in plasma at 37°C. 100 μl of aliquots were removed from each plasma and transferred to already STI (soybean trypsin inhibitor) containing tubes at every 15 min intervals. The samples were centrifuged for 10 min at 4000 rpm and the supernatant collected. To supernatant, 900 μl of sodium sulfite (10.5% stock concentration) was added and incubated for 15 min at 37°C, followed by centrifugation at 4000 rpm for 15 min. The supernatant was discarded and 400 μl of sodium sulfite was added and vortexed after breaking the pellet. The mixture was centrifuged at 4000 rpm for 15 min. Supernatant was discarded and pellet was dissolved in 300 μl of 4M Urea containing 0.1N NaOH. The mixture was incubated for 15 min at 100°C. Then, the pellet was dissolved by vigorous shaking and absorbance of the suitably diluted samples was taken at 280 nm. Estimation of fibrinogen was done in duplicates [38, 39].

### Determination of plasma half-life of nSK and PEGylated SK

The study on animals was carried out strictly in accordance with the ethical guidelines of the Institute (Institute of Microbial Technology) and approved by Institutional Animal Ethics Committee (IAEC) vide Approval number IAEC/11/5. *In vivo* half-life of the nSK or PEGylated constructs was determined in CD1 mice by injecting 50 μg of these proteins, in duplicates. Prior to injecting into mice, all the above proteins preparations were passed through a column of Polymyxin B Agarose (BioRad Inc., Palo Alto, CA, USA) [40] gel for removing endo-toxins. CD1 mice, each weighing 23–25 gm were anaesthetized with 3% iso-fluorane and mild vasodilation was induced by exposing the tail to a 100-watt fluorescence lamp. Around 50 μg protein in sterile phosphate buffer saline (PBS) was injected via the tail vein into the mice. Approximately 50 μl of whole blood samples were collected at different time intervals by tail transection or from retro-orbital sinus, and transferred to heparinized eppendorf tubes. After the last collection, mice were sacrificed by cervical dislocation. The samples were centrifuged at 2000 g for 10 min to recover plasma which was further incubated after suitable dilution with 20 nM plasmin in an assay buffer containing 0.5% bovine serum albumin, 50 mM Tris-HCl buffer (pH 7.5), 1 μM plasminogen and 1 mM Chromozyme ^®^PL in a 96-well plate. The residual levels of various constructs in plasma were determined indirectly from a reference curve prepared by plotting activity per time^2^ vs varying amount of the standards [35, 36].

### Immune-reactivity of nSK and its PEGylated-SK constructs

The relative immunogenicity of nSK or PEGylated-SK constructs was examined using nSK anti-sera (polyclonal) raised in rabbits by an ELISA-based method, as described previously [41, 32]. Firstly, nSK was diluted in 0.05 M bicarbonate buffer, pH 9.2 to obtain 0.015 μg to 0.030 μg final concentration of the protein in a 100 ul solution and this was added to each well of the microtiter plate (Nunc 96-Well Microplates, Cole-Parmer USA). These (nSK) antigen-coated plates were then incubated overnight at 4°C. Next day, the plates were washed twice with wash buffer comprising of phosphate buffered saline (PBS) containing 0.05% tween 20. The unoccupied sites were blocked with 200 μl of blocking buffer containing 10% skim milk in PBS for 2 h at room temperature (22°C) followed by three times washing with the wash buffer. Appropriate dilution of nSK/PEG-SK proteins were mixed with primary antibody solution for competition and were incubated for 30 min at 37°C in a mixture of 1 μM NPGB (*p-*nitrophenyl *p-*guanidinobenzoate) and 0.15 ml skimmed milk. All the protein dilutions were done in PBS. 100 μl of the above diluted primary antibody (1:5,000) was added to each well. The plate was incubated at room temperature for 1 h. Thereafter, the plate was again washed three times with the wash buffer and subsequently 100 μl of an appropriately diluted horse-radish peroxidase enzyme-labeled secondary antibody (1:10,000 in PBS), was added into each well. The plate was then incubated at room temperature for 1 h and washed three times with wash buffer. To each well 100 μl of 1X TMB (Tetramethylbenzidine Liquid substrate (Sigma-Aldrich, USA) was added and the plate incubated for 30 min at room temperature. The reaction was stopped by adding 50 μl 1.0 N sulphuric acid to each well and the color intensity was recorded spectrophotometrically at 450 nm [32].

### PEGylation of nSK cysteine mutants

Cysteine mutants of nSK were PEGylated using maleimide-activated linear methoxy PEG [42] of 10KDa or 20KDa (JenKem Technology, USA). For the PEGylation reaction, 2 mg protein was kept in 100 mM HEPES buffer, pH 6.0 containing 2 mM EDTA and 0.5 mM TCEP (tris(2-carboxyethyl) phosphine). To this, 9.2 mg of either 10KDa or 18.2 mg of 20KDa PEG reagent was added. The reaction mix was allowed to gently stir at room temperature for 6–12 h (standardized for every reaction).The reaction was stopped by addition of 1 mM of DTT. Alternatively, for N-terminal PEGylation, The protein was reduced with 6 M GITC (Guanidinium thiocyanate) and 1 mM DTT prior to PEGylation [43]. PEGylated protein was separated from free PEG by anion exchange chromatography on a DEAE-Sepharose™ column (GE-Healthcare) at 4°C. For this, the reaction mixture was diluted 10–15 times with 2 mM HEPES buffer (pH 7.0) and loaded onto a pre-equilibrated column of DEAE-Sepharose (Fast Flow). The column was washed with a 2mM HEPES buffer pH 7.0 buffer and the bound protein was eluted using a linear salt gradient (0–0.5 M NaCl) in 2 mM HEPES buffer. Subsequently, the eluted protein was subjected to size-exclusion chromatography on Sephacryl S-100 column (Amersham Biosciences) in buffer comprising 100 mM HEPES buffer (pH 6.0), 2 mM EDTA, 150 mM NaCl and 10 mM DTT, to separate unPEGylated SK from PEGylated form.

## Results

### Construction, purification and PEGylation of nSK variants

**Fig 1:**
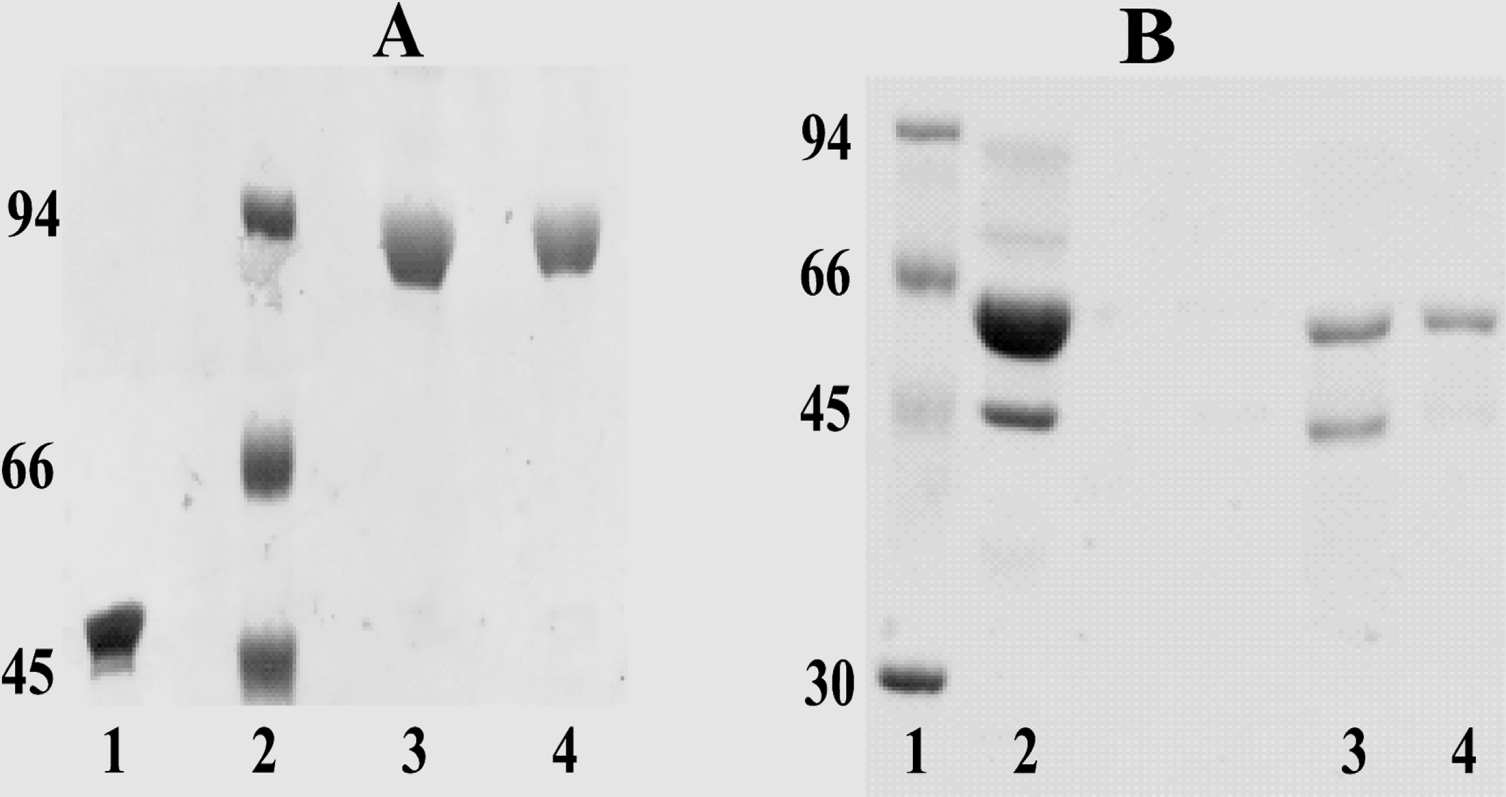
SDS-PAGE analysis of the SK-PEGylated protein. **(A)** SDS-PAGE analysis of purified SK1-383C-PEG20 (C-terminal truncated cysteine mutant PEGylated with 20KDa moiety) derivative. Lane 1: unpegylated protein; lane 2: molecular weight markers; lanes 3 and 4: Fractions 1 and 2 of purified SK1-383C-PEG20, through gel filtration chromatography. **(B)** Electrophoresis of SK1-383N-PEG10 (N-terminal cysteine mutant PEGylated with 10KDa moiety) SK derivative stained with coomassie. Lane 1: molecular weight markers; lane 2: PEGylation reaction mixture; lane 3: PEGylated protein after anion-exchange chromatography; lane 4: PEGylated protein after gel filtration chromatography.

**Fig 2:**
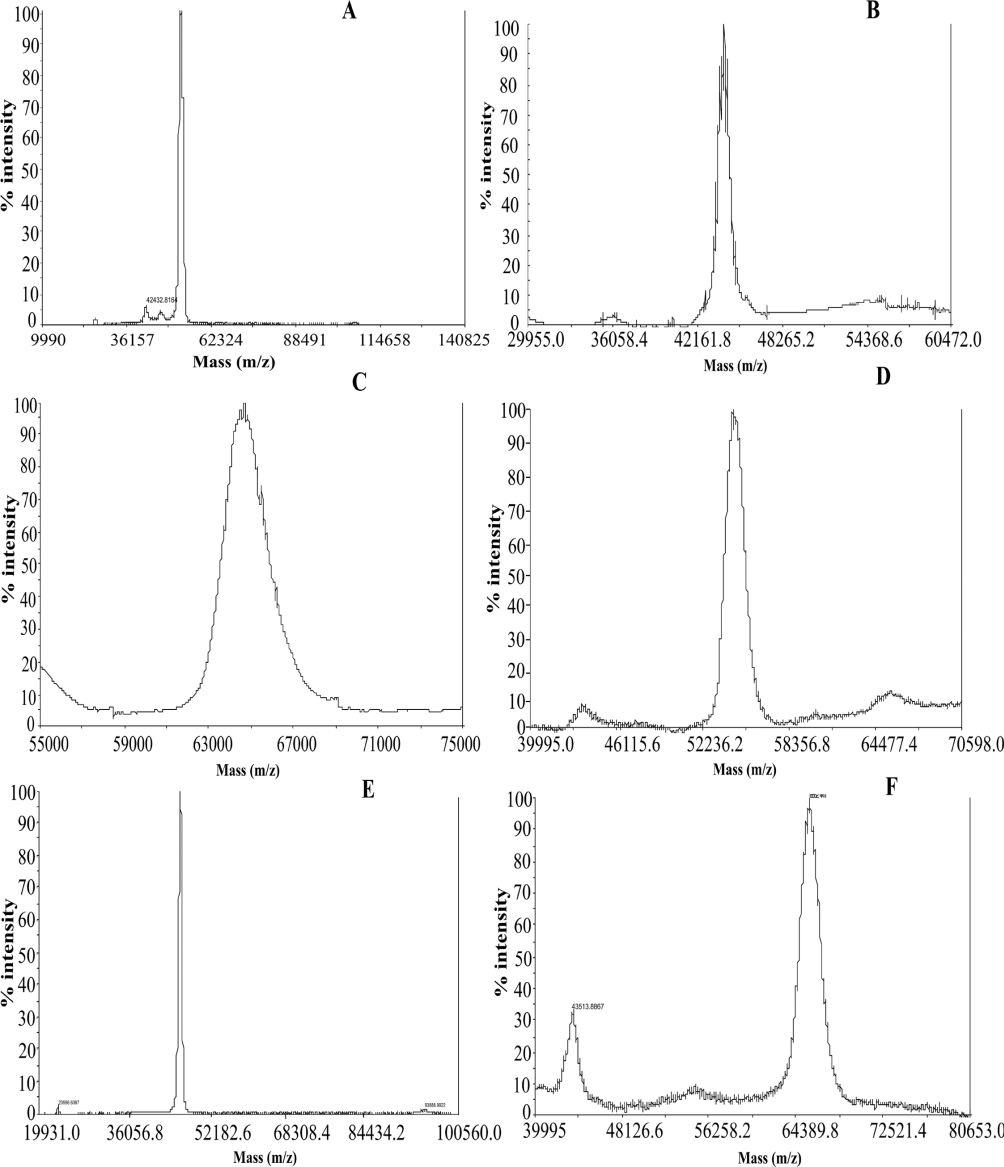
Determination of molecular weight of PEGylated derivatives by mass-spectrometry (MALDI-TOF). Molecular weight determined by MALDI-TOF of the PEGylated and unPEGylated-SK mutants were in agreement with their calculated molecular weight. **(A)** SK1-383N-PEG10 **(B)** SK1-383C-cys **(C)** SK1-383N-PEG20 **(D)** SK1-383C-PEG10 **(E)** nSK **(F)** SK1-383C-PEG20.

SK is totally bereft of cysteine [44] and hence in the present study, the site-specific PEGylation was performed in the cysteine-substituted SK constructs by methoxy-malaemide chemistry. SK-constructs which were truncated at either C or N-terminal were employed to generate uniform and site-specific PEGylated complexes [19, 43, 45]. Under the optimized condition, PEGylation of the mutated constructs at the terminal ends resulted in approximately 75–80% of PEGylated-SK complexes while 10–15% remained unPEGylated as observed on SDS-PAGE (Fig 1). The mono-PEGylated derivatives were then enriched by a two-step purification process which comprised of an anion-exchange chromatography for removing the unbound PEG molecules, followed by gel-filtration chromatography for separating PEGylated SK complex from the unPEGylated protein. PEGylation of SK1-383C-PEG20 derivative (C-terminal truncated cysteine mutant PEGylated with 20KDa moiety) showed presence of PEGylated SK (at 92KDa) and small amount unPEGylated SK (45KDa) in 7.5% SDS-PAGE. The latter was subsequently removed by gel-filtration chromatography (Fig 1A, lanes 3 and 4). Likewise, the reaction of SK1-383N-cys (N-terminal truncated cysteine mutant) with 10KDa PEG molecule, resulted in PEGylated (60KDa) and the native unPEGylated-SK (44KDa) (Fig 1B, lane 2). The unreacted PEG and unconjugated-SK were separated from PEGylated-SK complex by anion-exchange and gel-filtration chromatography, respectively (Fig 1B, lanes 3 and 4). Incidently, the PEGylated-SK complex displayed anomalous migration on SDS-PAGE. Therefore, the exact molecular weight of the SK-PEG complexes was determined by MALDI-TOF analysis [46–47] and as evident from Fig 2 was in accord with the expected theoretical values.

### PEGylated-SK complex displayed plasmin-dependent activation of HPG as compared to nSK

**Fig 3:**
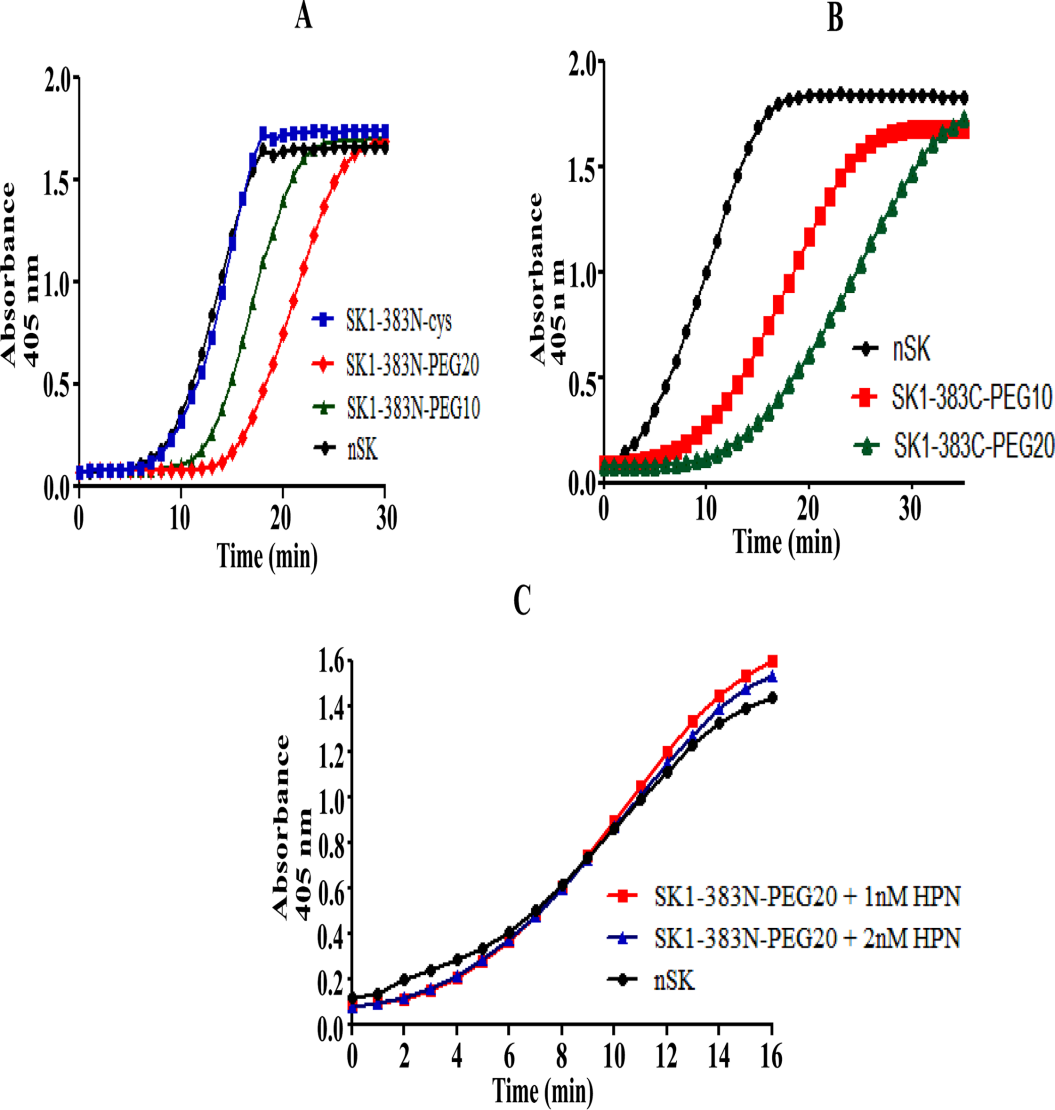
Depiction of time-course activation of HPG by nSK, truncated SK and PEGylated SK and PN-dependency of PEG-SK derivatives for the activation of HPG. **(A)** nSK/truncated SK/PEGylated-SK (0.5 nM conc. each) were added separately in microtiter plate wells, containing HPG and chromozyme^®^ PL. The progress curves of activation of HPG were measured spectrophotometrically at 405nm over time. The graph shows nSK (black color circles)SK1-383N-cys (blue rectangles), SK1-383N-PEG10 (green triangles), SK1-383N-PEG20 (red diamonds). **(B)** Graph represents activation of HPG by nSK/SK1-383C-PEG derivatives at 1 nM conc. each in the assay. nSK (black circles), SK1-383C-PEG10 (red rectangles), SK1-383C-PEG20 (green triangles). **(C)** Plasmin (1 nM or 2 nM) was incubated in a well containing nSK/truncated PEG-SK derivative, HPG and chromozyme ^®^ PL. Graph depicts native SK (black circles), SK1-383N-PEG20 containing 1 nM HPN (red rectangles), SK1-383N-PEG20 containing 2 nM HPN (blue triangles). Statistical analysis showed significant difference (p-value < 0.0001, n = 10, one-way ANOVA test).

A one-stage activity was performed to determine the rates of activation of HPG by PEGylated-SK constructs and nSK. The PEGylated derivatives of nSK were incubated with HPG and Chromozyme ^®^ PL in a 96-well plate and the time-course of HPG activation was monitored by recording absorbance at 405 nm [5, 35]. PEGylated-SK constructs exhibited a distinctly more prolonged lag in plasminogen activation as compared to nSK, which under the experimental conditions showed a remarkably shorter lag-period of about 7 min (Fig 3). Moreover, with increase in the molecular weight of PEG-conjugated to SK, the HPG activation was perceptibly protracted (Fig 3A).

For instance, SK1-383N-PEG20 (red diamonds) exhibited a lag-period of 14 min as compared to 11 min for SK1-383N-PEG10 (green triangles)(Fig 3A). The duration of HPG-activation period for un-PEGylated-SK cysteine mutants (blue rectangles) was identical to that for nSK (black circles). As with the N-terminal PEGylated derivatives, C-terminal SK-PEGylated constructs also exhibited a longer lag-period for the HPG-activation which was exaggerated with increase in the size of PEG-moiety. The, SK1-383C-PEG10 (red rectangles) and SK1-383C-PEG20 (green triangles) displayed HPG-activation period of 7 and 12 min, respectively, as compared to about 4 min for nSK (black circles) (Fig 3B). Evidently, PEGylation of SK at either terminal retarded the HPG activation though to different extents. Furthermore, the biological activity of different PEGylated-SK constructs was compared with the native SK. Two different concentrations of plasmin, viz., 1 and 2 nM, were incubated with PEGylated-SK (PEGylated at either N or C-terminal), HPG and Chromozyme. It is noteworthy that in the presence of plasmin both the PEGylated-SK constructs demonstrated HPG-activation that was comparable with that of nSK. It can thus be inferred that N-terminal PEGylated-SK constructs i.e. SK1-383N-PEG10 and SK1-383N-PEG20 follow Pathway 2 of HPG activation and their activation is accelerated in presence of plasmin, which is generally prevalent around the clot.

### Amidolytic activation of HPG/μPG by nSK/PEG-SK variants at 37°C and 4°C

**Fig 4:**
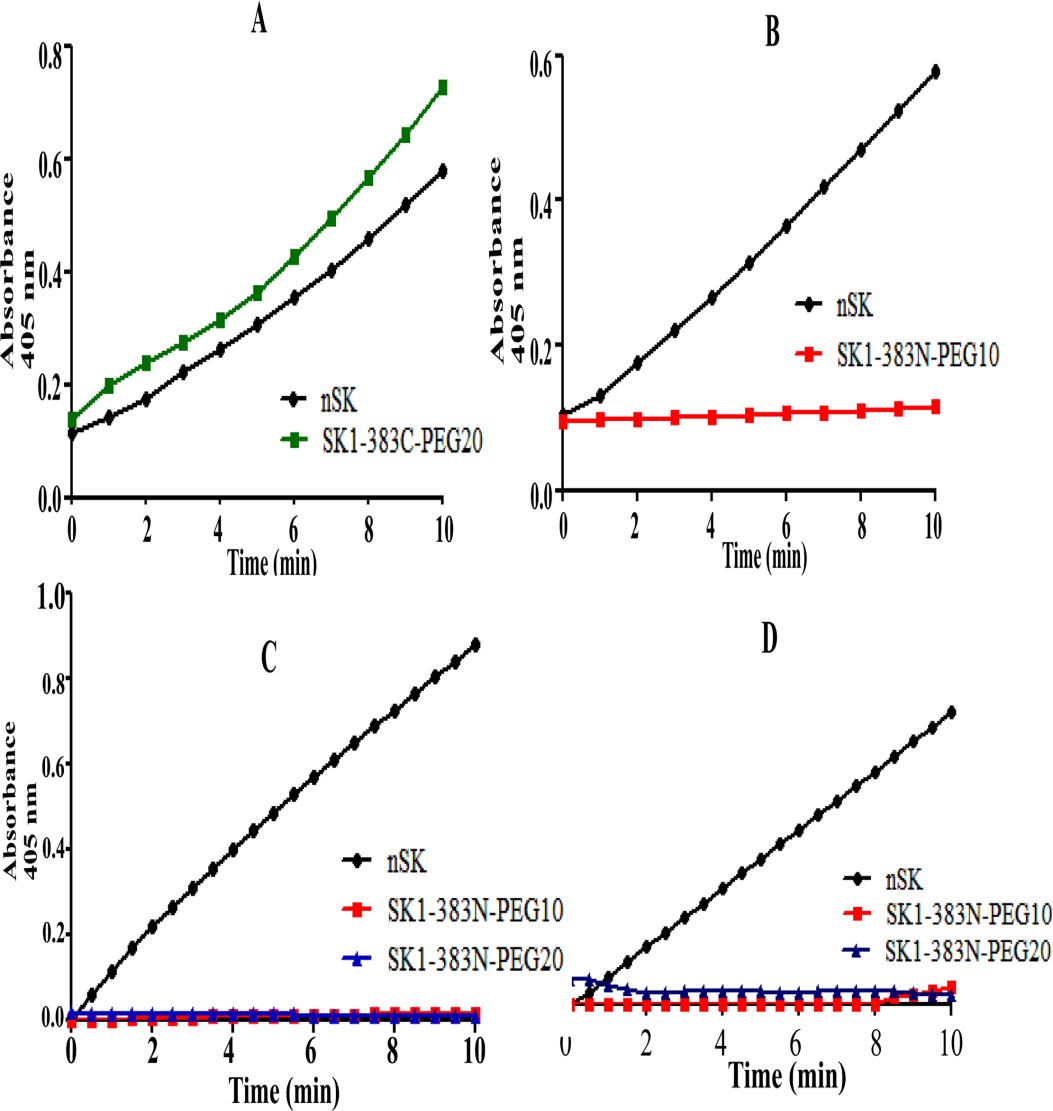
Amidolytic activity of nSK/truncated PEG-SK complexes with HPG/μPG at 37°C and 4°C. Pre-incubated HPG/uPG with nSK/truncated PEG-SK derivatives were added into the cuvette containing chromozyme ^®^ PL. Amidolytic activity generation were monitored by change in absorbance at A_405_ by hydrolysis of the substrate S-2251. **(A)** Graph displays amidolytic activation by native SK (black circles) and SK1-383C-PEG20 (green rectangles) in complex with μPG at 37°C. **(B)** Graph depicts the effect of conjugation of PEG moiety at N-terminal of derivative SK1-383N-PEG10 (red rectangles) by blocking its amidolytic activation in comparison with nSK (black circles), after making complex with μPG, at 37°C. **(C)** Graph displays the blocking of pathway I by N-terminally PEGylated SK truncated derivatives i.e. SK1-383N-PEG10 (red rectangles), SK1-383N-PEG20 (blue triangles) in comparison with nSK (black circles), when in complex with uPG, at 4°C. **(D)** Graph depicts no amidolytic activation by SK1-383N-PEG10 (red rectangles) and SK1-383N-PEG20 (blue triangles) in comparison with nSK (black circles), after making 1:1 complex with HPG, at 4°C. Statistical analysis showed significant difference (p-value < 0.0001, n = 6, one-way ANOVA test).

**Fig 5:**
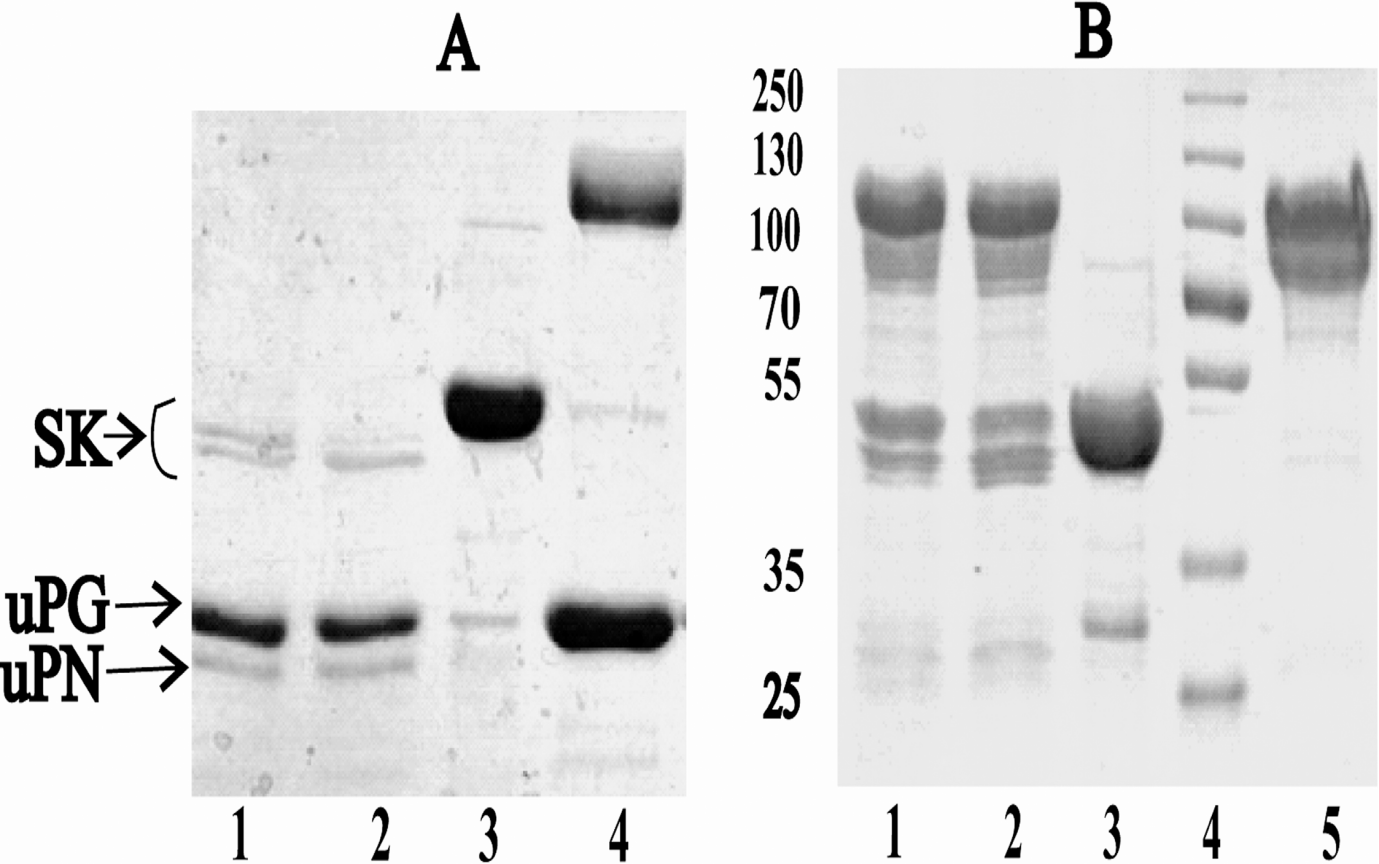
SDS-PAGE analysis of the HPG/μPG-nSK/PEG-SK complexes. Native SK/PEG-SK was incubated with HPG/μPG to form 1:1 complex at 5 μM concentration. After 3 and 10 min, each of these complexes were subjected to SDS-PAGE under reducing conditions. **(A)** Gel demonstrates the fragmentation of nSK after complex formation with μPG while no effect was noticed with PEG-SK derivatives. Lane 1: nSK-μPG complex (3 min); lane 2: nSK-μPG complex (10 min); lane 3: Streptokinase alone; lane 4: SK1-383N-PEG20-μPG (10 min). **(B)** SDS-PAGE indicates the blockage of pathway I when in complex with HPG. Lane 1: nSK-HPG complex (3 min); lane 2: nSK-HPG complex (10 min); lane 3: Streptokinase alone; lane 4: Molecular weight marker; lane 5: SK1-383N-PEG20-HPG (10 min).

The amidolytic activity of truncated-SK-PEGylated constructs and nSK as their respective complexes with HPG and μPG was monitored at A_405_ nm at both 37°C and 4°C [5]. At 37°C, as with nSK (Fig 4A, black circles), a perceptible, though somewhat feeble, μPG-mediated activation was observed for SK1-383C-PEG20 (green rectangles) but not for the SK1-383N-PEG10-μPG complex (Fig 4B, red rectangles) under identical experimental conditions. Unlike nSK (black circles), SK1-383N-PEG10 (red rectangles) and SK1-383N-PEG20 (blue triangles) in complex with μPG (Fig 4C) and HPG (Fig 4D), did not show any detectable activity at 4°C. Furthermore, the amidolytic activity of nSK and truncated-SK-PEGylated variants in complex with HPG or μPG was also examined at 37°C and 4°C for different time-intervals by SDS-PAGE analysis (Fig 5). In 7.5% SDS-PAGE, different intermediate fragments of nSK and μPN/HPN were observed in both nSK-uPG (Fig 5A, lane 1) and nSK-HPG complexes (Fig 5B, lane 1) within 3 min of complex formation. It is clearly seen that the main band of nSK (47 KDa) fragmented into two bands within 3 min of complex formation and which (upper band) further fragmented into smaller fragments after 10 min. Similarly, μPG (28 KDa) disintegrated into μPN within 3 min of complex formation. Moreover, nSK was completely cleaved into smaller fragments in both nSK-μPG (Fig 5A, lane 2) and nSK-HPG complexes (Fig 5B, lane 2) after 10 min. Under the same experimental conditions, no fragmentation of nSK (negative control) was observed in the absence of μPG (Fig 5A and 5B, lane 3). Importantly, SK1-383N-PEG20-μPG and SK1-383N-PEG20-HPG complexes did not undergo fragmentation even after 10 min of complex formation. Therefore, it can be inferred that PEGylation prevents fragmentation in nSK. These results, as shown in Figs 4 and 5, evidently signify that C-terminal PEGylation did not impede the HPG or μPG activation at 37°C in the truncated PEGylated-SK complexes, while, in contrast, the N-terminal PEGylation completely abolished this activity.

### PEG-SK constructs showed clot-lysis activity comparable to nSK

**Fig 6:**
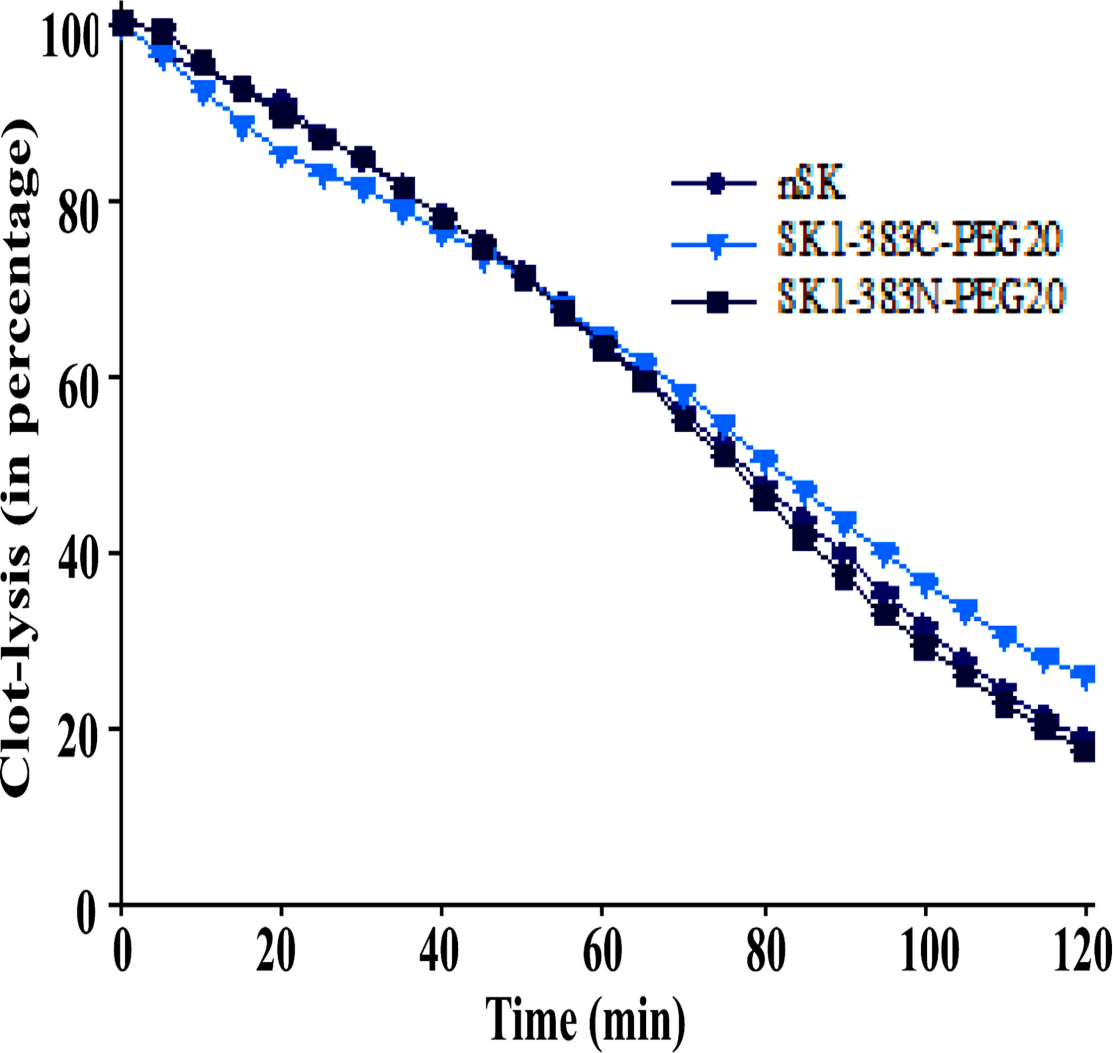
Demonstration of clot-lysis profile of PEG-SK variants in comparison with nSK at 5 nM concentration. A 100 μl of clot was incubated with nSK or its PEGylated derivatives in a microtitre plate at 37°C. Lysis of the clot was measured with time at A_405_ nm on ELISA plate reader. Graph represents the lysis of clot by SK1-383N-PEG20 (dark blue rectangles) and SK1-383C-PEG20 (blue triangles) in comparison with nSK (black circles). Experiment was performed in triplicates and standard deviation is shown with error bars.

The clot-lysis activity of nSK and truncated SK-PEGylated constructs was ascertained by the conventional approach in a microtitre plate. Initially, fibrin clot was formed by reconstituting fibrinogen, thrombin and plasminogen in a Tris-HCl buffer, and the potency of nSK and truncated SK-PEGylated constructs to lyse the clots at various time-points was evaluated by recording absorbance at A_405_ nm [37]. From Fig 6, it is apparent that the truncated SK-PEGylated constructs (both at N- and C-termini) as well as nSK, were equally effective in clot-lysis. At 5 nM concentration, SK1-383N-PEG20 (dark blue rectangles) and SK1-383C-PEG20 (blue triangles), exhibited near-identical time-course profiles as that of nSK (black circles). This is indicated by the observation that the truncated SK-PEGylated constructs manifested 50% clot-lysis at the same time (i.e. 75 min) as shown by nSK.

### PEGylated-SK constructs exhibited decreased fibrinogenolysis activity as compared with nSK

The impact on PEGylated-SK constructs as compared to nSK, on the fibrinogen lysis in blood, was evaluated by plasma fibrinogenolysis assay by using sodium-sulfite method [37]. nSK and PEGylated-SK constructs (SK1-383C-PEG20 and SK1-383N-PEG20) were incubated at 25 nM concentration with human plasma at 37°C, and aliquots were collected after every 15 min, and analyzed for residual fibrinogen levels. It is evident from the data in Table 1 that at 25 nM, SK1-383C-PEG20 containing wells had approximately 80 and 65%, after 15 and 30 min of the initial fibrinogen levels, while SK1-383N-PEG20 displayed 93 and 85% fibrinogen levels as compared to the corresponding levels of around 66% and 45% for nSK.

**Table 1:** Comparison of the presence of fibrinogen (in %) in plasma in presence of native SK and PEG-SK variants^a^.

Time points	Concentration (nM)	nSK (%)	SK1-383C-PEG20 (%)	SK1-383N-PEG20 (%)
15 min	25.0	65.9	81.2	93.0
30 min	25.0	44.8	66.7	83.8

^a^25 nM concentration of nSK/truncated PEGylated derivatives was incubated in plasma and appropriate amount of aliquots were removed after every 15 min to determine the amount of fibrinogen (see Materials and Methods for details). Average values (mean of three determinations) of variants are shown in the table.

**Fig 7:**
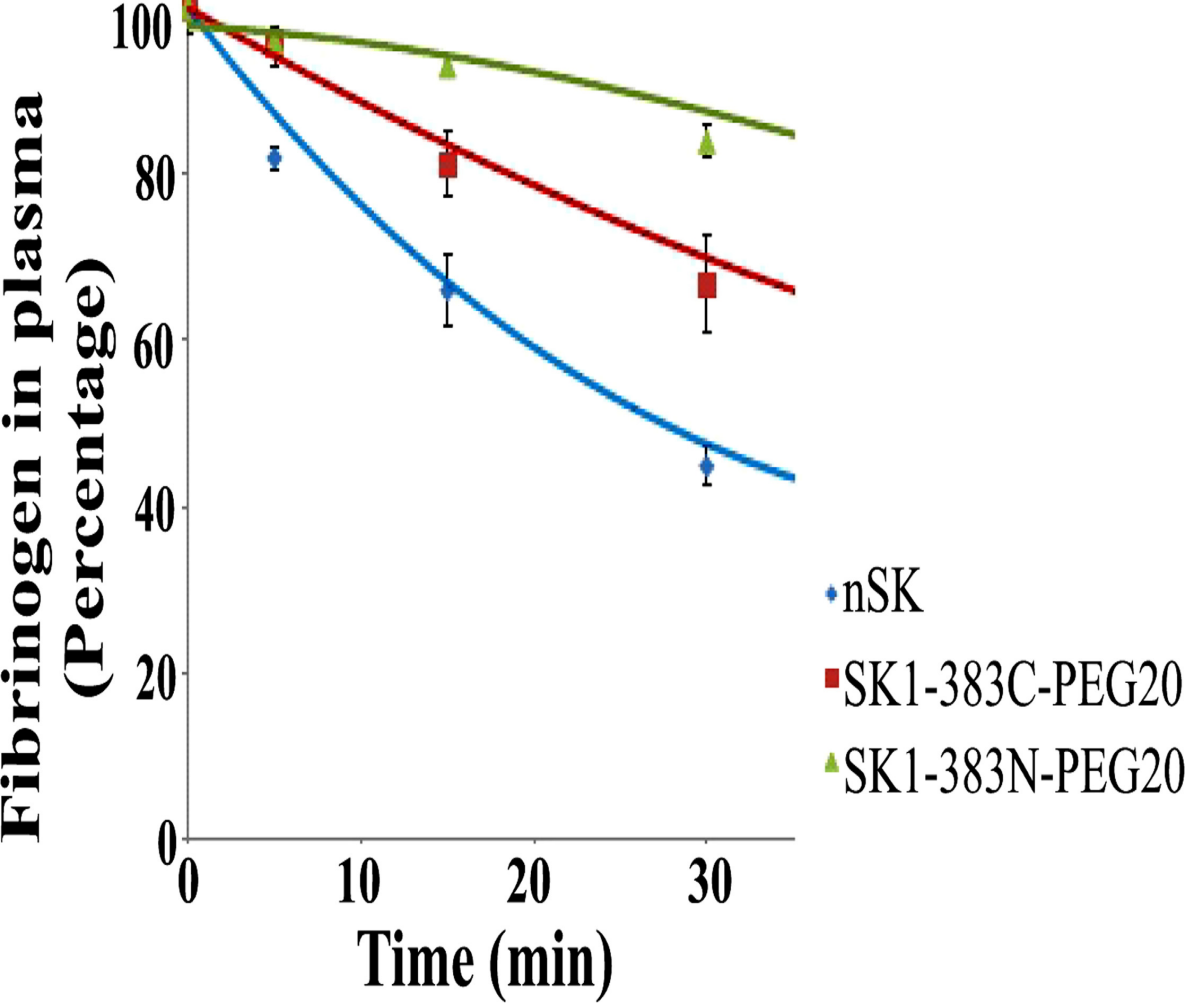
Determination of fibrinogen (in percentage) in the presence of native SK and PEG-SK variants in plasma. 25 nM concentration of nSK/truncated PEGylated derivatives were incubated with plasma and suitable volume of aliquots were removed after every 10 min for determination of the residual fibrinogen content. Graph represents the rate of fibrinogen degradation (in percentage) with time at 25 nM concentration by nSK (blue diamonds), SK1-383N-PEG20 (green triangles) and SK1-383C-PEG20 (red rectangles). Experiment was performed in triplicates and standard deviation is shown with error bars.

These results indicate that 50% of fibrinogen was degraded in about 30 min by nSK at 25 nM (Fig 7, blue diamonds), and tends to suggest that it would have taken about 1 h and >1 h at the same concentration of SK1-383N-PEG20 (Fig 7, green triangles) and SK1-383C-PEG20 (Fig 7, red rectangles) to reach the same levels, clearly showing a protective influence of PEGylation.

### PEGylation enhances plasma half-life of SK

The plasma (*in vivo*) half-life of truncated PEGylated-SK constructs and nSK were assessed by injecting 50 μg of the different constructs in mice and their activity was monitored in plasma at different time intervals. The drained plasma-containing nSK or PEGylated SK constructs samples from the mice were incubated with plasmin and Chromozyme in a 96-well plate and absorbance was recorded at 405 nm. The residual levels of various constructs in plasma were indirectly determined from reference curve/s that was constructed by plotting activity per time^2^ v/s varying amount of the standards. The PEGylated-SK constructs at C-terminal (SK1-383C-PEG10 and SK1-383C-PEG20) exhibited plasma half-life of 7.5 h and ~9 h, respectively (Table 2).

**Table 2:** Comparison of half-life of truncated PEG-SK derivatives with nSK in plasma^a^.

Derivatives	Half-life
nSK	15–20 min
SK1-383C-PEG10	7.5 h
SK1-383C-PEG20	>9 h
SK1-383N-PEG10	1 h
SK1-383N-PEG20	2 h

^a^50 μg of nSK/truncated PEGylated derivatives were intravenously administered in mice and their half-live were measured by PN-dependent activation assay (see Materials and Methods for details). Average values (mean of three determinations) of variants are shown above.

The N-terminal PEGylated-SK constructs i.e. (SK1-383N-PEG10 and SK1-383N-PEG20) disappeared more rapidly with half-lives of 1 h and 2 h, respectively (Table 2). The half-life of nSK was merely 15–20 min under the same conditions. It is evident that C-terminal PEGylation of SK yielded preparations with considerably longer plasma half-life compared to the corresponding N-terminal constructs.

### PEGylated-SK constructs demonstrated lower immune-reactivity compared to nSK

**Fig 8:**
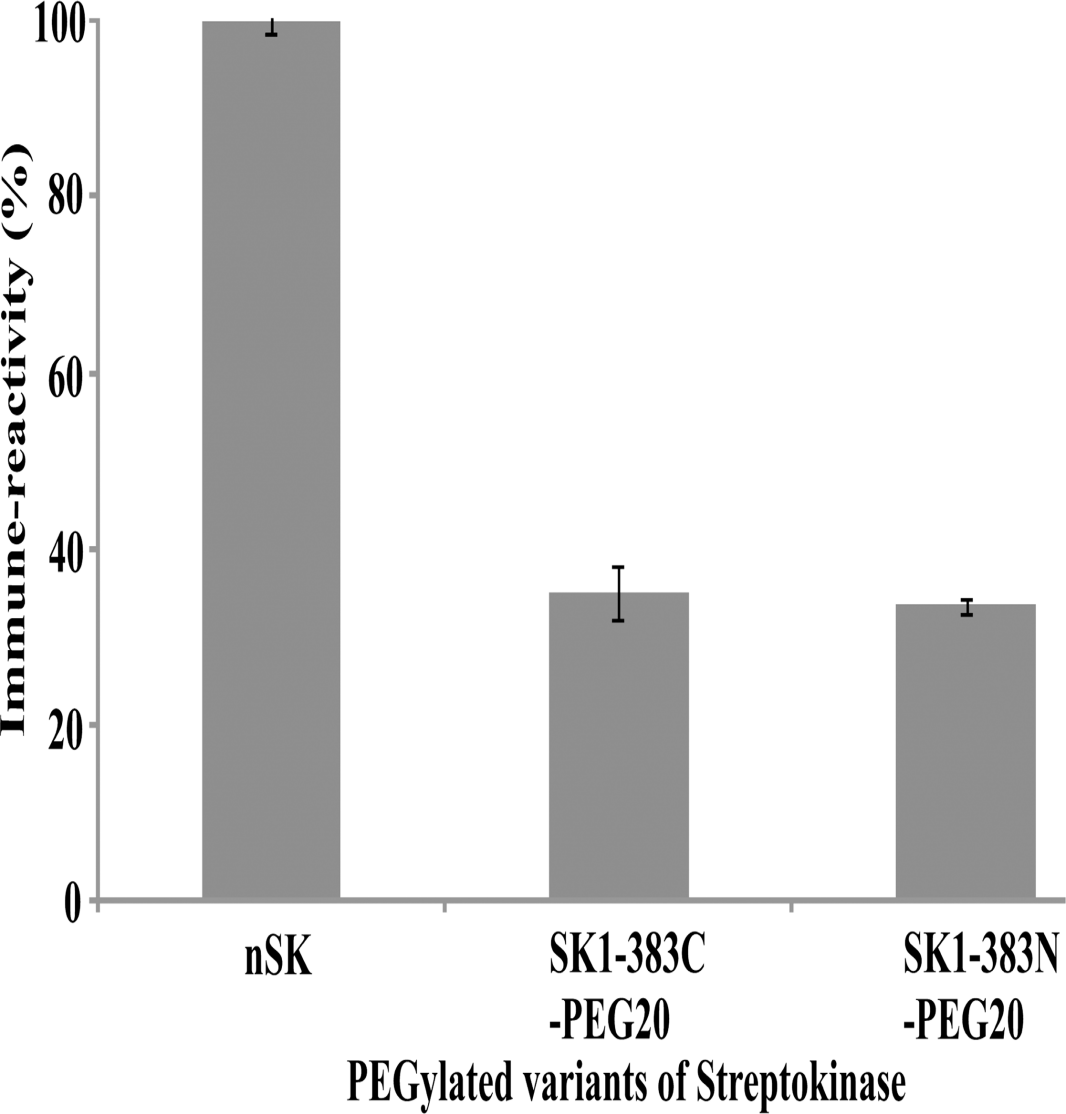
Demonstration of the relative reactivity of PEGylated SK truncated variants with rabbit polysera. The reactivity of nSK and PEGylated SK truncated variants were determined against rabbit polysera and expressed as percentage relative to that displayed by the former (taken as 100 percent reactivity). ELISA plates were coated with nSK and competition against polysera by PEGylated and unPEGylated (indicated on abscissa of each graph) proteins were assessed separately (see Materials and Methods section). Experiment was performed in triplicates and standard deviation is shown with error bars.

The immune-reactivity of PEGylated-SK constructs and nSK was determined by competitive enzyme-linked immunosorbent assay (ELISA) using polysera obtained from rabbits immunized with nSK. Results in Fig 8 indicate that, at equivalent protein concentrations, the PEGylated-SK constructs, namely SK1-383C-PEG20 and SK1-383N-PEG20, elicited 65% and 67% lower immune reactivity as compared to nSK.

## Discussion

Streptokinase is a highly effective thrombolytic agent, which, being relatively more economical is often used in less affluent societies for myocardial infarction. However, if some of its major drawbacks can be rectified, it has the potential to occupy centre-stage as an effective thrombolytic, especially for ischemic strokes. Some drawbacks which invite attention are systemic activation of HPG [48] and the consequent fibrinogen depletion by SK, which, if rectified, it can be given as a bolus shot instead of a slow infusion that requires hospital settings; another objective has been to reduce SK’s antigenicity as much as possible, while increasing its *in vivo* half-life. It has been earlier shown that PEGylation of SK considerably enhances its therapeutic attributes [30, 49] but an uncontrolled (non-specific) PEGylation is likely to lead to non-homogenous populations of PEGylated-SK adducts with varying physiochemical, pharmaceutical and biological properties [30]. A more rigorously controlled, site-specific PEGylation of SK will facilitate production of preparations having superior consistency and clinical reproducibility. Besides, it will enable designing of more reliable, concise and convenient strategies for removal of unreacted materials and impurities during production. In the present investigation, site-specific PEGylation of SK was attempted after cysteine incorporation through maleimide chemistry [42]. PEG groups of two different molecular masses *viz*. 10 KDa and 20 KDa were conjugated to the cysteine mutants of N-and C-terminally truncated-SK constructs. It has been previously shown that N-terminal and C-terminal truncated constructs are biologically active [17–19].

The PEGylated-SK complexes were evaluated for various functional attributes to assess their thrombolytic efficacy. The results obtained indicate that PEGylated-SK1-383C construct displayed HPG activation similar to that of the nSK molecule. In contrast, under the same experimental conditions, no HPG activation was observed for the N-terminally modified construct, namely PEGylated-SK1-383N-PEG. The inability of the N-terminal PEGylated SK construct in activating HPG could be attributed to the fact that N-terminal of SK contains a ''catalytic switch'' which imparts it an ability to activate PG both via fibrin-dependent and fibrin-independent mechanisms [50]. In addition, Ile at the first position at N-terminal is involved in formation of a complex with HPG which results in catalytic activation and conversion of " free" HPG to HPN [16]. In contrast, the C-terminally truncated-SK, SK1-383C-Cys, upon PEGylation with either 10 KDa or 20 KDa, bears an intact original N-terminus, and showed, as expected, an intact Pathway 1 activation capability. Incidentally, both the N- and C- terminally PEG-modified constructs displayed almost the same level of activity as the native SK in the presence of plasmin wherein Pathway 2 is operative.

The kinetics of non-proteolytic zymogenic or amidolytic activation of both N- and C-terminally PEGylated SK1-383 constructs were then examined by SDS-PAGE patterns at 37°C and 4°C, respectively. In conformity with the results obtained for the HPG activation assay, the kinetic analysis also indicated that PEGylated-SK1-383C possessed activity almost identical to native-SK. On the other hand, PEGylated-SK1-383N constructs, irrespective of the PEG size used for conjugation, were completely devoid of HPG activation. The obtained results signify that the activation of HPG by PEGylated-SK1-383N constructs entails formation of a complex that consists exclusively of SK1-383N-PEG and PN rather than the complex of SK1-383N-PEG and PG. Plasmin has previously been shown to be released in high concentrations in the vicinity of clots which leads to a fast conversion of substrate HPG into HPN for fibrinolysis in the blood clots [51, 52]. Our studies denote that PEGylated-SK1-383N construct would be more efficient and act as a fibrin specific plasminogen activator as it will become active only in the presence of plasmin, present in the fibrin clots and not that in the general circulation, which lacks free plasmin.

The clot lysis activity of the PEGylated-SK constructs was also assessed. It is relevant that both the N-terminus and C-terminus constructs PEGylated either with 10 KDa or 20 KDa moiety displayed almost same rate of degeneration of fibrin clots with time as seen with nSK. The specificity of both N-and C-terminally PEGylated SK1-383 constructs was estimated by determining the relative amount of fibrinogen in plasma and compared with nSK. Both N-and C-terminally PEGylated SK1-383 constructs exhibited markedly lower fibrinogen depletion in plasma than with nSK. The results are in accord with the previous report about fibrinogen depletion by nSK [53, 54]. Hence, these constructs are promising fibrinolytic molecules as they are highly fibrin-specific and do not contribute to systemic activation of HPG to plasmin which often leads to hemorrhagic and bleeding complications.

Significantly, besides maintaining an *in vitro* efficient clot-lysis activity and improved specificity, the *in vivo* half-life of PEGylated-SK1-383C-PEG constructs was drastically prolonged followed by PEGylated-SK1-383N-PEG constructs, a phenomenon that may be ascribed to the probable masking of highly sensitive proteolytically labile sites at the N-terminal region of SK which normally render it extremely susceptible to rapid cleavage. It is also pertinent that the truncated PEGylated-SK derivatives (either at C- or N-terminal) exerted a considerably diminished immune reactivity against rabbit polysera as compared with nSK. Thus, the results obtained in the present study regarding efficient clot-lysis activity and clot-specificity, specifically, the prolongation of *in vivo* half-life and a curtailed immune reactivity are extremely encouraging from the view point of new thrombolytic development. These also unmistakably suggest that some of the vital therapeutic attributes of this clot-dissolving agent can be successfully altered by a simple and selective chemical modification of truncated SK molecule/s using a protein engineering approach.

## Conclusion

The present study demonstrates the formulation of an efficient thrombolytic drug decorated with PEG moieties that posses desirable attributes of an enhanced *in vivo* half-life, reduced immune-reactivity and clot-specificity. Advantageously, these fibrin-specific clot-dissolving agents avert systemic activation of HPG in circulation, and therefore enhance the effectiveness of drug for the treatment of various circulatory diseases.

## References

[1] Tillett WS, Garner RL. The Fibrinolytic Activity of Hemolytic Streptococci. J Exp Med. 1933;58:485–502. 19870210 10.1084/jem.58.4.485 PMC2132310

[2] Collen D. Coronary thrombolysis: streptokinase or recombinant tissue-type plasminogen activator? Annals of internal medicine. 1990;112(7):529–38. 2107781 10.7326/0003-4819-112-7-529

[3] Sikri N, Bardia A. A history of streptokinase use in acute myocardial infarction. Texas Heart Institute Journal. 2007;34(3):318 17948083 PMC1995058

[4] Reddy KNN, Markus G. Mechanism of activation of human plasminogen by streptokinase Presence of active center in streptokinase-plasminogen complex. Journal of Biological Chemistry. 1972;247(6):1683–91. 4258976

[5] Aneja R, Datt M, Singh B, Kumar S, Sahni G. Identification of a new exosite involved in catalytic turnover by the streptokinase-plasmin activator complex during human plasminogen activation. Journal of Biological Chemistry. 2009;284(47):32642–50. 10.1074/jbc.M109.046573 19801674 PMC2781679

[6] Yadav S, Aneja R, Kumar P, Datt M, Sinha S, Sahni G. Identification through combinatorial random and rational mutagenesis of a substrate-interacting exosite in the gamma domain of streptokinase. J Biol Chem. 2011;286:6458–69. 10.1074/jbc.M110.152355 21169351 PMC3057801

[7] Chaudhary A, Vasudha S, Rajagopal K, Komath SS, Garg N, Yadav M, et al Function of the central domain of streptokinase in substrate plasminogen docking and processing revealed by site-directed mutagenesis. Protein Sci. 1999;8:2791–805. 10631997 10.1110/ps.8.12.2791 PMC2144232

[8] Sundram V, Nanda JS, Rajagopal K, Dhar J, Chaudhary A, Sahni G. Domain truncation studies reveal that the streptokinase-plasmin activator complex utilizes long range protein- protein interactions with macromolecular substrate to maximize catalytic turnover. J Biol Chem. 2003;278:30569–77. 12773528 10.1074/jbc.M303799200

[9] Dhar J, Pande AH, Sundram V, Nanda JS, Mande SC, Sahni G. Involvement of a nine-residue loop of streptokinase in the generation of macromolecular substrate specificity by the activator complex through interaction with substrate kringle domains. J Biol Chem. 2002;277:13257–67. 11821385 10.1074/jbc.M108422200

[10] Nihalani D, Kumar R, Rajagopal K, Sahni G. Role of the amino-terminal region of streptokinase in the generation of a fully functional plasminogen activator complex probed with synthetic peptides. Protein Sci. 1998;7:637–48. 9541396 10.1002/pro.5560070313 PMC2143961

[11] Reed GL, Lin LF, Parhami-Seren B, Kussie P. Identification of a plasminogen binding region in streptokinase that is necessary for the creation of a functional streptokinase-plasminogen activator complex. Biochemistry. 1995;34:10266–71. 7640282 10.1021/bi00032a021

[12] Wang X, Lin X, Loy JA, Tang J, Zhang XC. Crystal structure of the catalytic domain of human plasmin complexed with streptokinase. Science. 1998;281:1662–5. 9733510 10.1126/science.281.5383.1662

[13] Schick LA, Castellino FJ. Interaction of streptokinase and rabbit plasminogen. Biochemistry. 1973;12(22):4315–21. 4270765 10.1021/bi00746a003

[14] Boxrud PD, Bock PE. Coupling of conformational and proteolytic activation in the kinetic mechanism of plasminogen activation by streptokinase. J Biol Chem. [Research Support, U.S. Gov't, P.H.S.]. 2004 8 27;279(35):36642–9. 15215239 10.1074/jbc.M405265200

[15] Boxrud PD, Fay WP, Bock PE. Streptokinase binds to human plasmin with high affinity, perturbs the plasmin active site, and induces expression of a substrate recognition exosite for plasminogen. J Biol Chem. 2000;275:14579–89. 10799544 10.1074/jbc.275.19.14579

[16] Wang S, Reed GL, Hedstrom L. Deletion of Ile1 changes the mechanism of streptokinase: evidence for the molecular sexuality hypothesis. Biochemistry. 1999;38:5232–40. 10213631 10.1021/bi981915h

[17] Shi G-Y, Chang B-I, Chen S-M, Wu D-H, Wu H-L. Function of streptokinase fragments in plasminogen activation. Biochem J. 1994;304:235–41. 7998939 10.1042/bj3040235 PMC1137477

[18] Kim IC, Kim JS, Lee SH, Byun SM. C-terminal peptide of streptokinase, Met369-Pro373, is important in plasminogen activation. IUBMB Life. 1996;40(5):939–45. 10.1080/15216549600201563 8955883

[19] Zhai P, Wakeham N, Loy JA, Zhang XC. Functional roles of streptokinase C-terminal flexible peptide in active site formation and substrate recognition in plasminogen activation. Biochemistry. 2003;42(1):114–20. 12515545 10.1021/bi026746m

[20] Houng A, Quen S, Jean L, REED G, editors. Construction of a recombinant streptokinase that resists cleavage and inactivation by plasmin. Thrombosis and Haemostasis. 1995;73(6):1130.

[21] Schellenberger V, Wang C-w, Geething NC, Spink BJ, Campbell A, To W, et al A recombinant polypeptide extends the *in vivo* half-life of peptides and proteins in a tunable manner. Nat Biotechnol. 2009;27(12):1186–90. 10.1038/nbt.1588 19915550

[22] Ikuta S, Chuang VTG, Ishima Y, Nakajou K, Furukawa M, Watanabe H, et al Albumin fusion of thioredoxin—the production and evaluation of its biological activity for potential therapeutic applications. Journal of Controlled Release. 2010;147(1):17–23. 10.1016/j.jconrel.2010.05.020 20678999

[23] Subramanian GM, Fiscella M, Lamousé-Smith A, Zeuzem S, McHutchison JG. Albinterferon α-2b: a genetic fusion protein for the treatment of chronic hepatitis C. Nat Biotechnol. 2007;25(12):1411–9. 18066038 10.1038/nbt1364

[24] Duncan R. The dawning era of polymer therapeutics. Nature Reviews Drug Discovery. 2003;2(5):347–60. 12750738 10.1038/nrd1088

[25] Harris JM, Chess RB. Effect of pegylation on pharmaceuticals. Nature Reviews Drug Discovery. 2003;2(3):214–21. 12612647 10.1038/nrd1033

[26] Veronese FM, Mero A. The impact of PEGylation on biological therapies. BioDrugs. 2008;22(5):315–29. 18778113 10.2165/00063030-200822050-00004

[27] Bailon P, Won C-Y. PEG-modified biopharmaceuticals. 2009. 10.1517/17425240802650568 19236204

[28] Pasut G, Veronese FM. State of the art in PEGylation: the great versatility achieved after forty years of research. Journal of Controlled Release. 2012;161(2):461–72. 10.1016/j.jconrel.2011.10.037 22094104

[29] Alconcel SN, Baas AS, Maynard HD. FDA-approved poly (ethylene glycol)–protein conjugate drugs. Polymer Chemistry. 2011;2(7):1442–8.

[30] Rajagopalan S, Gonias SL, Pizzo SV. A nonantigenic covalent streptokinase-polyethylene glycol complex with plasminogen activator function. Journal of Clinical Investigation. 1985;75(2):413 3156148 10.1172/JCI111715 PMC423508

[31] Kumar S, Maheshwari N, Sahni G. Mutants of streptokinase and their covalently modified forms. 2012; United States patent US 8,093,032.

[32] Sawhney P, Kumar S, Maheshwari N, et al Site-Specific Thiol-mediated PEGylation of Streptokinase Leads to Improved Properties with Clinical Potential. Current Pharmaceutical Design (in press). 10.2174/1381612822666160204120547 26845325

[33] Deutsch DG, Mertz ET. Plasminogen: purification from human plasma by affinity chromatography. Science. 1970;170:1095–6. 5475635 10.1126/science.170.3962.1095

[34] Bradford M. M. Rapid and sensitive method for the quantitation of microgram quantities of protein utilizing the principle of protein-dye binding. Anal. Biochem. 1976; 72: 248–254. 942051 10.1016/0003-2697(76)90527-3

[35] Wohl RC, Summaria L, Robbins KC. Kinetics of activation of human plasminogen by different activator species at pH 7.4 and 37 degrees C. J Biol Chem. 1980;255:2005–13. 7188769

[36] Castellino FJ, Sodetz JM, Brockway WJ, Siefring GE. [21] Streptokinase. Methods Enzymol 1976;45:244–57. 1011996 10.1016/s0076-6879(76)45024-3

[37] Silva M, Thelwell C, Williams S, Longstaff C. Regulation of fibrinolysis by C-terminal lysines operates through plasminogen and plasmin but not tissue-type plasminogen activator. Journal of Thrombosis and Haemostasis. 2012;10(11):2354–60. 10.1111/j.1538-7836.2012.04925.x 22974122

[38] Mackie IJ, Kitchen S, Machin SJ, Lowe G. Guidelines on fibrinogen assays. British journal of haematology. 2003;121(3):396–404. 12716361 10.1046/j.1365-2141.2003.04256.x

[39] Rampling M, Gaffney P. The sulphite precipitation method for fibrinogen measurement; its use on small samples in the presence of fibrinogen degradation products. Clinica Chimica Acta. 1976;67(1):43–52. 10.1016/0009-8981(76)90215-1 1253452

[40] Morrison DC, Jacobs DM. Binding of polymyxin B to the lipid A portion of bacterial lipopolysaccharides. Immunochemistry. 1976;13(10):813–8. 187544 10.1016/0019-2791(76)90181-6

[41] Engvall E, Perlmann P. Enzyme-linked immunosorbent assay (ELISA) quantitative assay of immunoglobulin G. Immunochemistry. 1971;8(9):871–4. 5135623 10.1016/0019-2791(71)90454-x

[42] Gregory JD. The stability of N-ethylmaleimide and its reaction with sulfhydryl groups. J Am Chem Soc. 1955;77(14):3922–3.

[43] Veronese FM, Mero A, Caboi F, Sergi M, Marongiu C, Pasut G. Site-specific pegylation of G-CSF by reversible denaturation. Bioconjugate chemistry. 2007;18(6):1824–30. 17941684 10.1021/bc070123+

[44] Jackson KW, Tang J. Complete amino acid sequence of streptokinase and its homology with serine proteases. Biochemistry. 1982;21:6620–5. 6760891 10.1021/bi00269a001

[45] Kim DM, Lee SJ, Yoon SK, Byun SM. Specificity role of the streptokinase C-terminal domain in plasminogen activation. Biochem Biophys Res Commun. 2002;290:585–8. 11779212 10.1006/bbrc.2001.6238

[46] Lee H, Jang IH, Ryu SH, Park TG. N-terminal site-specific mono-PEGylation of epidermal growth factor. Pharmaceutical research. 2003;20(5):818–25. 12751640 10.1023/a:1023402123119

[47] Seyfried BK, Siekmann J, Belgacem O, Wenzel RJ, Turecek PL, Allmaier G. MALDI linear TOF mass spectrometry of PEGylated (glyco) proteins. Journal of Mass Spectrometry. 2010;45(6):612–7. 10.1002/jms.1746 20527029

[48] Keramati M, Arabi Mianroodi R, Memarnejadian A, Mirzaie A, Sazvari S, Mehdi Aslani M, et al Towards A Superior Streptokinase for Fibrinolytic Therapy of Vascular Thrombosis. Cardiovascular & Hematological Agents in Medicinal Chemistry (Formerly Current Medicinal Chemistry-Cardiovascular & Hematological Agents). 2013;11(3):218–29. 10.2174/187152571103140120103816 23531210

[49] Jevševar S, Kunstelj M, Porekar VG. PEGylation of therapeutic proteins. Biotechnology journal. 2010;5(1):113–28. 10.1002/biot.200900218 20069580

[50] Reed GL, Houng AK, Liu L, Parhami-Seren B, Matsueda LH, Wang S, et al A catalytic switch and the conversion of streptokinase to a fibrin-targeted plasminogen activator. Proceedings of the National Academy of Sciences. 1999;96(16):8879–83. 10.1073/pnas.96.16.8879 PMC17701 10430864

[51] Gaffney PJ. Molecular aspects of fibrin clot solubilization. Nature. 1971;234(52):281–2. 10.1038/newbio234281a0 4257271

[52] Marder VJ, Francis CW. PLASMIN DEGRADATION OF CROSS-LINKED FIBRIN*. Annals of the New York Academy of Sciences. 1983;408(1):397–406. 6223558 10.1111/j.1749-6632.1983.tb23260.x

[53] Green J, Harris G, Smith R, Dupe R. Acyl-enzymes: a novel class of thrombolytic agents. Thrombolysis:Biological and Therapeutic Properties of New Thrombolytic Agents, Churchill Livingstone, Edinburgh. 1985:124–67.

[54] Rao AK, Pratt C, Berke A, Jaffe A, Ockene I, Schreiber TL, et al Thrombolysis in Myocardial Infarction (TIMI) Trial—phase I: hemorrhagic manifestations and changes in plasma fibrinogen and the fibrinolytic system in patients treated with recombinant tissue plasminogen activator and streptokinase. Journal of the American College of Cardiology. 1988;11(1):1–11. 3121710 10.1016/0735-1097(88)90158-1

